# Robotic Liver Resection Versus Percutaneous Ablation for Early HCC: Short- and Long-Term Results

**DOI:** 10.3390/cancers12123578

**Published:** 2020-11-30

**Authors:** Paolo Magistri, Barbara Catellani, Samuele Frassoni, Cristiano Guidetti, Tiziana Olivieri, Giacomo Assirati, Cristian Caporali, Annarita Pecchi, Valentina Serra, Roberto Ballarin, Gian Piero Guerrini, Vincenzo Bagnardi, Stefano Di Sandro, Fabrizio Di Benedetto

**Affiliations:** 1 Hepato-Pancreato-Biliary Surgery and Liver Transplantation Unit, University of Modena and Reggio Emilia, 41124 Modena, Italy; paolo.magistri@unimore.it (P.M.); catellani.barbara@aou.mo.it (B.C.); cristianogui@yahoo.it (C.G.); tizianaolivieri3@gmail.com (T.O.); assirati.giacomo@aou.mo.it (G.A.); serra.valentina@aou.mo.it (V.S.); ballarin.roberto@aou.mo.it (R.B.); guerrini.gianpiero@aou.mo.it (G.P.G.); stefano.disandro@unimore.it (S.D.S.); 2 Department of Statistics and Quantitative Methods, University of Milan-Bicocca, 20126 Milan, Italy; samuele.frassoni@unimib.it (S.F.); vincenzo.bagnardi@unimib.it (V.B.); 3 Department of Radiology, Policlinico University Hospital of Modena, 41124 Modena, Italy; caporali.cristian@aou.mo.it (C.C.); annarita.pecchi@unimore.it (A.P.)

**Keywords:** hepatocellular carcinoma, robotic surgery, minimally invasive, survival, RFA, recurrence

## Abstract

**Simple Summary:**

Patients newly diagnosed with a single hepatocellular carcinoma (HCC) smaller than 3 cm that underwent robotic liver resection (RLR) benefit from a significantly higher potential of cure and tumor-related free survival compared to those that underwent percutaneous ablation (PA), with comparable incidence of morbidity. Notably, the 20.8% incidence of satellitosis in the RLR group confirms that PA patients are at higher risk to obtain a not-radical treatment and, therefore, are more likely to need repeated treatments, which actually doubles the risk of morbidity in the long term. Therefore, therapeutic algorithms as well as down-staging protocols for HCC can be implemented with robotic approaches in high volume centers with extensive experience in the field of hepato-biliary surgery and minimally invasive approaches, to offer more opportunities to those patients.

**Abstract:**

Background: The correct approach for early hepatocellular carcinoma (HCC) is debatable, since multiple options are currently available. Percutaneous ablation (PA) is associated in some series to reduced morbidity compared to liver resection (LR); therefore, minimally invasive surgery may play a significant role in this setting. Methods: All consecutive patients treated by robotic liver resection (RLR) or PA between January 2014 and October 2019 for a newly diagnosed single HCC, less than 3 cm in size (very early/early stages according to the Barcelona Clinic Liver Cancer (BCLC)) on chronic liver disease or liver cirrhosis, were enrolled in this retrospective study. The aim of this study was to compare short- and long-term outcomes to define the best approach in this specific cohort. Results: 60 patients fulfilled the inclusion criteria: 24 RLR and 36 PA. The two populations were homogeneous in terms of baseline characteristics. There were no statistically significant differences regarding the incidence of postoperative morbidity (RLR 38% vs. PA 19%, *p* = 0.15). The cumulative incidence of recurrence (CIR) was significantly higher in patients who underwent PA, with the one, two, and three years of CIR being 42%, 69%, and 73% in the PA group and 17%, 27%, and 27% in the RLR group, respectively. Conclusions: RLR provides a significantly higher potential of cure and tumor-related free survival in cases of newly diagnosed single HCCs smaller than 3 cm. Therefore, it can be considered as a first-line approach for the treatment of patients with those characteristics in high-volume centers with extensive experience in the field of hepatobiliary surgery and minimally invasive approaches.

## 1. Background

Hepatocellular carcinoma (HCC) accounts for about 90% of all primary liver malignancies with growing incidence on the global scale [1,2,3]. The management and expected outcomes of patients affected by HCC vary greatly according to the underlying liver disease and tumor stage. According to the Barcelona Clinic Liver Cancer (BCLC) algorithm, curative options for very early and early HCC include liver resection (LR), ablation (either radiofrequency (RFA) or microwave (MWA)), and liver transplantation (LT) [1]. Therefore, the correct approach for single tumors up to 3 cm is debatable, since multiple options are currently available. Evidence in the literature suggests that LR or RFA can provide comparable results for patients with single, small HCC and well-compensated liver disease, although recent reports show an advantage for LR over percutaneous ablation (PA) in terms of recurrence-free survival with comparable post-procedure morbidities [4]. However, whether or not ablation can achieve a similar outcome as LR for tumors within the 3-cm range remains unclear. In a case-control study, Vitali and colleagues showed that minimally invasive liver surgery offers a comparable overall and disease-free survival to percutaneous RFA in the management of selected patients with single HCC ≤ 3 cm, with both strategies having similar complication rates [2]. In this setting, laparoscopic and robotic surgery may play a major role in the treatment of very early and early HCC, thanks to the reduced morbidity related to minimally invasive liver resections [5]. In addition to the well-known advantages of minimally invasive approaches to the liver, robotic surgery may add further improvements thanks to its intrinsic characteristics—namely, magnified view, stability of the instruments and tremor filtration, and gentle tissue manipulation.

The aim of this study was to analyze the outcomes of patients who underwent robotic liver resection (RLR) or PA for newly diagnosed single HCC up to 3 cm in size, comparing short- and long-term outcomes, to define the best approach in this specific cohort.

## 2. Results

Two hundred and eighty-eight patients were treated for HCC with either surgical approach (130 cases) or PA (158 cases) from January 2014 to October 2019; in addition, 15 patients were treated by laparoscopic radiofrequency ablation. In detail, 78 patients received an LR with a robotic approach in the study period. Finally, we enrolled 60 patients that fulfilled the inclusion criteria: 24 RLR and 36 PA.

### 2.1. Patient and HCC Characteristics

No statistically significant differences were found between the two groups (Table 1). Patients treated by either PA or RLR had no differences in terms of age, sex, body mass index (BMI), American Society of Anesthesiologists (ASA) score, and Charlson Comorbidiy Index (CCI). Hepatitis C (HCV) infection represented the most frequent cause of chronic liver disease, accounting for 53.3% of the cases of the entire cohort. It was followed by hepatitis B (HBV), alcohol-related cirrhosis, and non-alcoholic steatohepatitis (NASH). One patient in the PA group and three patients in the RLR group showed human immunodeficiency virus (HIV) coinfection.

Furthermore, the two groups showed no statistically significant differences in terms of the Ishak fibrosis score (*p* = 0.4) and the presence of clinically significant portal hypertension (64% in the PA group and 58% in the RLR, respectively, *p* = 0.79), as reported in Table 2. The liver functional reserve was similar in the two groups according to MELD score (Model for End-stage Liver Disease) and CTP (Child-Turcotte-Pugh) score, while the Albumin-Bilirubin (ALBI) score varied in a statistically significant fashion (*p* = 0.01).

**Figure 1:**
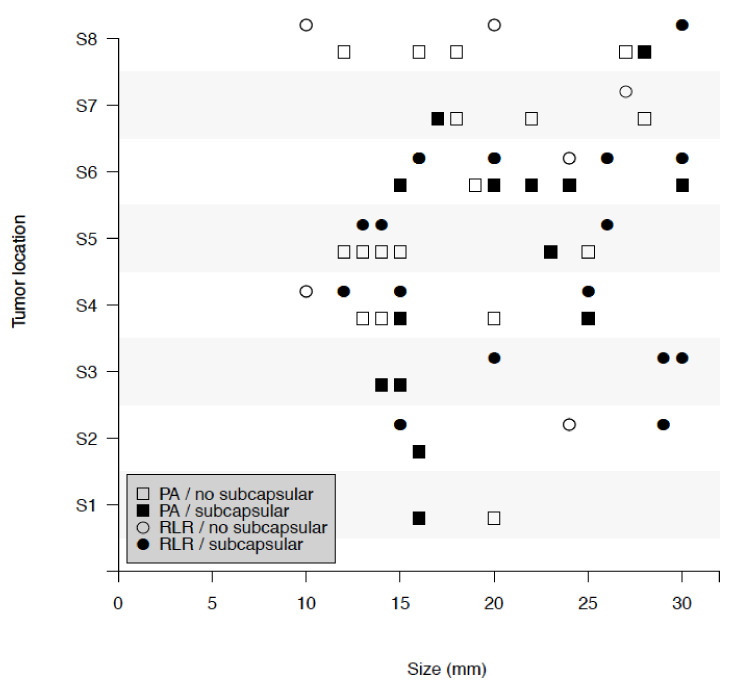
Tumor location and tumor size, among patients with percutaneous ablation (PA) and robotic liver resection (RLR) with and without subcapsular hepatocellular carcinoma (HCC).

The HCC size and alpha-fetoprotein (AFP) were similar between the two groups, even considering the cut-off of a 2-cm size. Regarding tumor location, the distribution of nodules in the hepatic segments was similar in the two groups (*p* = 0.25), while there was a higher prevalence of subcapsular nodules in the RLR group (75% RLR vs. 44% PA, *p* = 0.03) (see Figure 1). 

### 2.2. Procedure Characteristics

The RLR included 16 wedge resections and 8 segmentectomies, performed with or without intermittent hilar clamping (Pringle maneuver was performed in three cases: total clamping interval of 32, 50, and 72 min, respectively) and using a combination of monopolar energy, bipolar energy, and da Vinci Harmonic ACE for parenchymal transection. Hemostasis and biliostasis on the liver cut surface is achieved using either metallic clips, Hem-o-lok clips, nonabsorbable sutures, or a vascular stapler according to the size of the vessel. In nine cases, cholecystectomy was performed as a simultaneous procedure. No conversions to open surgery were required, and no intraoperative complications or deaths occurred. The median operative time (OT) was 200 min (range 70–380 min), while the median estimated blood loss (EBL) was 230 mL (range 10–800 mL). All RLR patients demonstrated a R0 resection, with a median distance from the margin of 10 mm (range 3–20). The histological examination showed 25% of well-differentiated (G1), 54% of moderately differentiated (G2), and 21% of poorly differentiated (G3) HCC, with the presence of satellitosis in 20.8% of cases and one case of microvascular invasion (Table S1).

No data regarding the operative time could be retrieved for percutaneous thermo-ablation in most of the cases (Table S2).

### 2.3. Short-Term Outcomes

**Figure 2:**
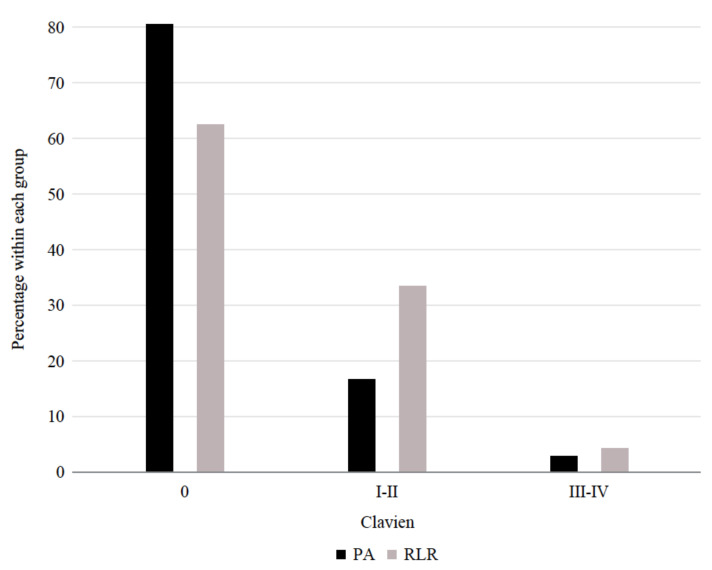
Distribution of the Clavien-Dindo morbidity among patients with PA and RLR.

Table 3 reports the outcomes of the procedures. The overall complications rate was not statistically different between the two groups (RLR 38% vs. PA 19%, *p* = 0.15). Six patients in the PA group experienced postprocedural complications grade I–II according to Clavien-Dindo (Figure 2). In detail, one patient developed gastroparesis, vomiting, and required naso-gastric tube (NGT) placement; three patients had a fever; one patient had a liver hematoma and fever; and one patient had a fever and high transaminase levels. Eight patients in the RLR had the same grade of complications: three patients with ascites, one patient developed a pneumothorax, two patients had fevers, one patient had mild pneumonia, and one patient suffered a grade B post-hepatectomy liver failure (PHLF) according to the International Study Group of Liver Surgery (ISGLS) definition (deviation from the regular clinical management but manageable without invasive treatment) [6]. Only one patient in each group developed a Clavien-Dindo grade III-IV complication: a post-procedure subcapsular hematoma and laceration that required arterial embolization and transfusion (PA) and an abdominal abscess (RLR).

Postoperative median bilirubin peak was 1.45 mg/dL in the PA group and 1.41 mg/dL in the RLR group, *p* = 0.59. Similarly, the international normalized ratio (INR) and creatinine peaks did not differ significantly. 

The median in-hospital stay after PA was shorter than that in the RLR group, two days versus four (ranges one–six and 1–18, respectively, *p* = < 0.001). It should be mentioned that only four patients that received RLR had a hospital stay longer than seven days. No reoperation and procedure-related 30-day mortality were observed; one readmission within 30 days from discharge occurred in the RLR group due to encephalopathy and two in the PA group, one for bowel obstruction and one for a liver hepatic abscess, treated conservatively with antibiotic therapy (*p* = 1). In Table 4, those results are also stratified by resection type.

### 2.4. HCC Recurrence and Survival

**Figure 3:**
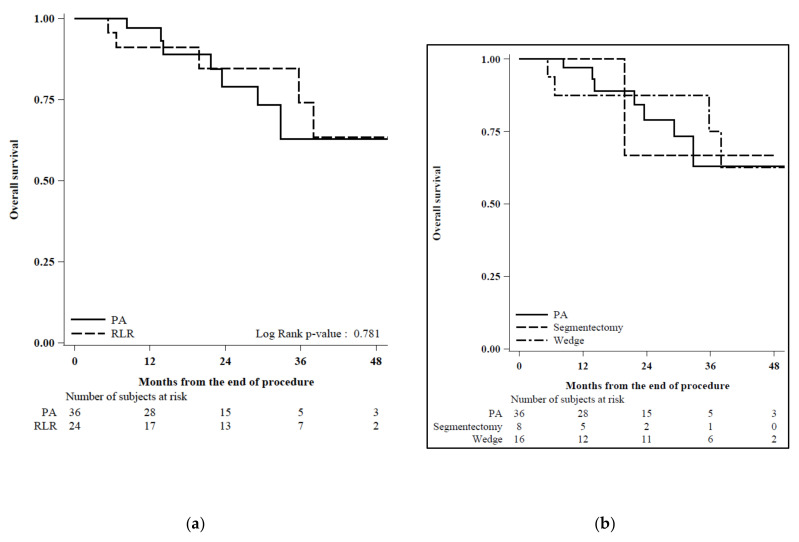
(**a**) Overall survival by procedure (N = 60): Observations were censored at the date of the transplant. Cause of deaths: N = 4 “HCC” and N = 3 “Others” in PA and N = 3 “HCC” and N = 2 “Others” in RLR. (**b**) Cause of deaths: N = 4 “HCC” and N = 3 “Others” in PA, N = 1 “Others” in Segmentectomy, N = 3 “HCC”, and N = 1 “Others” in Wedge.

**Figure 4:**
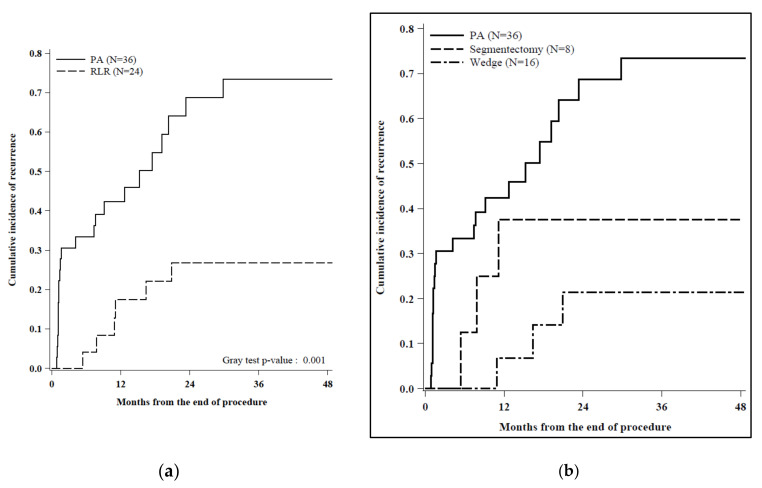
(**a**) Cumulative incidence of recurrence by procedure (*N* = 60): Recurrence as first event was considered as “Event”. Transplant or reoperation/locoregional treatment postresection or death as first event was considered as “Competing event”. No event at the last follow-up was considered as “Censored”. Of the 22 patients that experienced a recurrence in the PA group, 12 were perilesional and 10 were not, while all the 6 cases of recurrence in the RLR group were not perilesional. (**b**) Cumulative incidence of recurrence by procedure, stratified by the RLR type of resection: Recurrence as first event was considered as “Event”. Transplant or reoperation/locoregional treatment postresection or death as first event was considered as “Competing event”. No event at last FU was considered as “Censored”. Events: recurrence (*N* = 22 in PA (12 perilesional and 10 not perilesional), *N* = 3 in segmentectomy (3 not perilesional and *N* = 3 in Wedge (3 not perilesional)). Competing events: transplant (*N* = 1 in PA, *N* = 2 in segmentectomy, and *N* = 2 in Wedge) and death (*N* = 1 in PA, *N* = 1 in segmentectomy, and 2 in Wedge). Censored: no event at the last FU (*N* = 12 in PA, *N* = 2 in segmentectomy, and *N* = 9 in Wedge).

The median follow-up time in the PA group was 22 months (range 13–32) and 29 months in the RLR group (range 11–38). One-, two-, and three-year overall survival (OS) were 97%, 79%, and 63% in the PA group and 91%, 85%, and 74% in the RLR group, respectively (Figure 3), without reaching a statistically significant threshold (*p* = 0.78). Conversely, twenty-two events were observed during follow-up in the PA cohort (61%) versus six events in the RLR cohort (25%), determining a cumulative incidence of recurrence (CIR) significantly higher in patients who underwent PA, with one, two, and three years of CIR being 42%, 69%, and 73% in the PA group and 17%, 27%, and 27% in the RLR group, respectively (Figure 4).

The incidences of residual disease and all-site recurrence were also different between the two groups: of the 22 patients that experienced a recurrence in the PA group, 12 were perilesional and 10 were not, while all the 6 cases of recurrence in the RLR group were not perilesional. 

Patients with HCC progression were treated by Trans-catheter Arterial Chemo-Embolization (TACE) in 12 cases, TACE plus RFA in one case, MWA in two cases, and robotic liver resection in two cases. Finally, 15 patients in the study group underwent liver transplantation: seven patients in the RLR group and eight patients in the PA group.

In the RLR group, two deaths were related to HCC progression, one to non-surgery-related cirrhosis decompensation, and two to non-liver-related causes; in the PA group, four deaths were related to HCC progression, two to non-surgery-related cirrhosis decompensation, and one to non-liver-related causes.

Subgroup analyses for tumors smaller than 2 cm showed the same results of the general cohort (Tables S3 and S4).

## 3. Discussion 

Even if surgery still represents the gold standard treatment for patients with single HCC, it has been proposed that nonsurgical options, such as PA, may be considered as alternative primary treatments when the expected operative mortality rate is greater than 3% [7]. The reasons that may favor PA as a first-line treatment instead of surgical resection include the significantly lower risk of morbidity and related procedural costs associated with PA. On the other hand, RFA and MWA present major limitations, such as a lack of histological features of the nodule and inability to check the radicality of the ablation immediately.

Several studies suggested that patients with early HCC and well-compensated liver disease (CTP A and early CTP B) and hepatic vein pressure gradient (HVPG) below 10 mmHg can be safely treated by either minimally invasive surgery (MIS) or RFA [3,8]. In 2008, Livraghi et al. [9] reported that RFA was associated with lower complication rates and lower costs for patients with very early stage HCC (single tumor < 2 cm), with similar survival. In contrast, in a study by Liu et al. [10], LR was reported to be preferable over RFA in a similar population, with statistically significant better OS and recurrence-free survival (RFS). Moreover, for tumors measuring 21 to 30 mm, data in the literature are controversial; LR is generally associated to better long-term survival compared with PA and worse perioperative outcomes, with higher postprocedural morbidity and mortality. However, over the last decade, thanks to the spread of minimally invasive surgery for HCC on liver cirrhosis, a reduction of the perioperative complication rate was observed, particularly in terms of liver failure, bleeding, and hospital stay [11,12,13]. Moreover, the minimally invasive approach proved to be particularly effective in reducing the risk of PHLF in patients with clinically significant portal hypertension (CSPH) [14]. Taken together, these data suggest that minimally invasive liver surgery may represent a stand-alone alternative to other approaches in the BCLC flow-chart, increasing the chance of getting a more radical treatment and reducing the risk of postoperative complications. 

In this setting, the robotic approach may represent a novel player thanks to its additional advantages over standard laparoscopy. A magnified stable view and increased dexterity allow for a more precise parenchymal transection, reducing the risk of postoperative morbidity [15]. Moreover, even difficult segments [16,17] can be safely approached with the robotic approach, as demonstrated in our cohort that included three segment VIII and one segment VII resections. 

This study has some limits. Given its retrospective nature, the decision to perform PA or RLR was not the result of a randomization process but was taken according to clinical considerations, as reported above. For example, the RLR group included more cases of subcapsular tumors, which are at high risk of rupture in the case of PA. However, the two cohorts were ultimately well-balanced, and no statistically significant differences could be found in terms of age, ASA score, CTP score, and MELD. In fact, even if the number of patients with bilirubin >1 was significantly higher in the PA group; the CTP and MELD scores were comparable among the two populations, confirming that the overall performance status was not different.

Besides the longer median in-hospital stay, our experience demonstrates that RLR is not related to increased morbidity compared to PA and provides better long-term outcomes thanks to a reduced risk of recurrence. In this regard, the 21.74% incidence of satellitosis in the RLR confirms that PA is at a higher risk to obtain a nonradical treatment. Therefore, RLR may be considered as a first-line approach for newly diagnosed very early and early HCCs in centers that have reasonable experience with minimally invasive liver surgery [18,19]. However, the main goal is to offer to each patient the most appropriate treatment according to the individual’s performance status and comorbidities, instead of offering the same approach indiscriminately. We herein provide evidence that RLR is capable of offering radical resections, providing longer recurrence-free survival, without a significant increase of postprocedural complications. Moreover, the OS was similar between the RLR and PA groups, but patients in the PA group were more likely to need repeated treatments, which actually doubled the risk of morbidity in the long term. PA remains a milestone in the treatment of HCC; however, it should be probably reserved for cases that present specific contraindications or refuse the surgical approach.

## 4. Material and Methods

### 4.1. Study Design

All consecutive patients treated by RLR or PA between January 2014 and October 2019 for a newly diagnosed single HCC, less than 3 cm in size (very early/early stages according to BCLC [1]) on chronic liver disease or liver cirrhosis, were enrolled in this retrospective study. Data were obtained from our prospectively collected institutional databases. A subgroup analysis for tumor sizes less than 2 cm (≤2 cm) was performed.

HCC diagnosis was based on the European and American guidelines (EASL and AASLD) [1,7] and discussed in our multidisciplinary meeting, including surgeons, hepatologists, diagnostic radiologists, and interventional radiologists to define the optimal approach [20]. Preoperative ultrasound scan (US) of the abdomen, triphasic computed tomography (CT) scan, and contrast-enhanced magnetic resonance (MR) are part of the staging workup and are performed according to the multidisciplinary board advice and patient-specific condition. Both CTP A and CTP B patients and noncirrhotic liver diseases are considered eligible for robotic resection. Macrovascular tumor invasion and general contraindications to maintain a pneumoperitoneum (respiratory diseases or anesthesiologic concerns) are currently considered contraindications to RLR in our center. On the other hand, peripheral exophytic lesions, tumors close to major branches of the portal vein or hepatic veins, macrovascular tumor invasion, and nodules undetectable by US are contraindications to PA. Therefore, patients with exophytic lesions, tumors close the hepatic hilum or major hepatic veins, and nodules undetectable by US were excluded from this analysis. After surgery, all patients were followed-up at our surgical outpatient clinic at variable intervals according to the specific disease. Follow up included clinical examination, liver function tests, and imaging according to the multidisciplinary consensus. The first CT scan or MRI is usually performed one month after treatment, with either PA or RLR; then, if the first check is negative, the follow-up continues with CT or MR every three months for the first two years and every six months thereafter. The study was approved by our Institutional Review Board (#187/14), was conducted according to the Helsinki Declaration principles, and patients agreed to use anonymous data for research purposes. 

### 4.2. Variables and Definitions

The study was performed according to the Strengthening the Reporting of Observational Studies in Epidemiology (STROBE) guidelines [21]. The study included variables concerning demographic and clinical characteristics: sex, age (years), body mass index (BMI, kg/m^2^), American Society of Anesthesiologists score (ASA), Charlson Comorbidiy Index (CCI) with 0 defined as “no comorbid conditions recorded”), co-pathology, and the etiology of liver disease. The following liver disease-related variables were collected: Ishak fibrosis score (F0: score of 0–4 indicating none-to-moderate fibrosis and F1: score of 5 to 6 indicating severe fibrosis or cirrhosis), presence of esophageal varices and serum levels of albumin (g/dL), total bilirubin (md/dL), creatinine (mg/dL), international normalized ratio (INR), PLT (103/mmc), and AFP (ng/mL). MELD score, CTP score, and ALBI score were calculated and reported. HCC data included the long-axis diameter (mm) and location (subcapsular node or not and hepatic segment involved). 

Clinically significant portal hypertension (CSPH) is defined as HVPG > 10 mmHg or platelet count < 100 associated with splenomegaly or the presence of esophageal varices.

For patients in the RLR group, the following intraoperative data were collected and analyzed: estimated blood loss (EBL, mL); operative time, including docking of the robotic cart (OT, minutes); pringle maneuver (number of cycles, times, and technique),;conversion to open approach; deaths; and other intraoperative procedures. 

For both groups, the length of hospital stay (LOS days); postprocedural peak levels of bilirubin, INR, and creatinine; postoperative complications graded using the Clavien-Dindo classification [22]; reinterventions; in-hospital mortality; and readmission within 30 days after the procedure were collected. The eighth edition of the American Joint Committee on Cancer (AJCC) Cancer Staging Manual was used to stage the disease at the histological evaluation.

Tumors were defined as “subcapsular” when localized below the Glissonian layer, located at <1 cm in depth from the liver surface and associated with a liver surface bulge, without an exophytic growth [4].

The primary endpoint was to compare the long-term treatment effectiveness and, therefore, the incidence of recurrence, defined as follows: 

Incomplete ablation: enhancement present at the ablation border on the arterial phase CT at 1 month and consistent with the residual tumor;

Local recurrence: an enhancing lesion contiguous with the ablation or resected zone that is present on subsequent imaging and was not present on the initial post-procedure scan; for RLR, local recurrence is defined as recurrence in the same liver segment after local excision, while, for percutaneous thermo-ablation, local recurrence is any recurrent lesion within 2 cm of ablation site;

Regional recurrence: hepatic recurrence that is not adjacent to the ablation site; and

Metastatic recurrence: extrahepatic recurrence, including lymph node metastases.

The secondary endpoint was to compare the two approaches in terms of the postprocedural incidence of morbidity.

### 4.3. Statistical Analysis 

Continuous data are reported as median and range; categorical data are reported as counts and percentages.

Comparisons between percutaneous ablation and robotic liver resection were performed using the Wilcoxon signed-rank test for continuous variables and Fisher’s exact test for categorical variables.

Overall survival (OS) was estimated using the Kaplan-Meier method, censoring the observation at the date of the transplant. The log-rank test was used to assess differences among procedures.

The cumulative incidence function (CIF) of recurrence was estimated according to the method described by Kalbfleisch and Prentice, taking into account the competing causes of recurrence (transplant and death). Gray’s test was used to assess differences among the procedures.

A *p*-value <0.05 was considered statistically significant in all the analyses. All the analyses were performed with the statistical software SAS 9.4 (SAS Institute, Cary, NC, USA) and R (R version 3.4.3, https://www.r-project.org/).

### 4.4. Robotic Liver Resection

RLRs were performed using the da Vinci Si robotic platform (Intuitive Surgical inc., Sunnyvale, CA, USA) by the same surgeon, with experience in hepatobiliary surgery, liver transplant, and minimally invasive surgery, who already completed his robotic learning curve [18]. Each procedure was performed on the basis of patient and tumor characteristics, as a result of accurate preoperative planning. Technical aspects of the RLR were already reported [23]. The decision to perform anatomical vs. non-anatomical resection was based on patient-specific liver reserves, which were determined as described above.

### 4.5. Percutaneous Ablation

All patients were placed under conscious sedation, and the procedure was carried out under local anesthesia with ultrasonographic guidance. Three senior interventional radiologists performed the procedures. 

Percutaneous RFA was performed using a RITA^R^ StarBurst XL–Electrosurgical Device (AngioDynamics Inc., Latham, NY, USA), which is a single electrode placed centrally within the HCC nodules. The radiofrequency is usually emitted for 10 to 15 min at 100 W; timing and energy varies according to the tumor size and location. The ablation process may be repeated to obtain an entire treated area of at least 1 cm around the edges of the nodule.

Percutaneous MWA was performed using a Solero–Microwave Tissute Ablation Applicator (AngioDynamics Inc., Latham, NY, USA) for 3–6 min at 60–80 W; timing and the energy issued varied according to the tumor size and location.

MWA is preferred when the lesion is close to a vessel at the preprocedural ultrasound study. In fact, MWA is related to a reduced heat “sink effect” compared to RFA. 

## 5. Conclusions

RLR provides a significantly higher potential of cure and tumor-related free survival in cases of newly diagnosed single HCCs smaller than 3 cm. Therefore, it can be considered as a first-line approach for the treatment of patients with those characteristics in high-volume centers with extensive experience in the field of hepatobiliary surgery and minimally invasive approaches when the appropriate learning curve is completed.

## Figures and Tables

**Table 1:** Demographic and clinical characteristics (*N* = 60).

Variable	Level	PA (*N* = 36)	RLR (*N* = 24)	*p*-Value ^1^
Age (years), median (min–max)		67 (20–87)	64 (48–79)	0.52
Age (years), *N* (%)	<75	25 (69)	21 (88)	0.13
	≥75	11 (31)	3 (13)	
Sex, *N* (%)	Male	26 (72)	16 (67)	0.78
	Female	10 (28)	8 (33)	
BMI (Kg/m^2^), median (min–max)		28.12 (17.82–38.20)	27.25 (19.82–33.03)	0.67
BMI (Kg/m^2^), *N* (%)	Underweight	1 (4)	0 (0)	0.90
	Normal	6 (26)	7 (29)	
	Overweight	8 (35)	10 (42)	
	Obese	8 (35)	7 (29)	
	Missing	13	0	
ASA, *N* (%)	I	4 (11)	0 (0)	0.13
	II	10 (28)	11 (46)	
	III	22 (61)	13 (54)	
CCI score, median (min–max)		7 (2–10)	7 (3–9)	0.62
Previous abdominal surgeries, *N* (%)	No	19 (53)	14 (58)	0.79
	Yes	17 (47)	10 (42)	
Hypertension, *N* (%)	No	20 (56)	8 (33)	0.12
	Yes	16 (44)	16 (67)	
Diabetes, *N* (%)	No	28 (78)	20 (83)	0.75
	Yes	8 (22)	4 (17)	
Renal failure, *N* (%)	No	36 (100)	22 (92)	0.16
	Yes	0 (0)	2 (8)	
Liver cirrhosis, *N* (%)	No	15 (42)	6 (25)	0.27
	Yes	21 (58)	18 (75)	
Respiratory insufficiency, *N* (%)	No	34 (94)	23 (96)	1.00
	Yes	2 (6)	1 (4)	
Cardiovascular disease, *N* (%)	No	29 (81)	21 (88)	0.73
	Yes	7 (19)	3 (13)	
Hepatopathy, *N* (%)	HCV	20 (56)	12 (50)	0.60
	HBV	8 (22)	6 (25)	
	Alcohol	3 (8)	2 (8)	
	NASH	4 (11)	1 (4)	
	HCV+HBV	1 (3)	1 (4)	
	CBP	0 (0)	2 (8)	
HIV coinfection, *N* (%)	No	35 (97)	21 (88)	0.29
	Yes	1 (3)	3 (13)	

ASA: American Society of Anesthesiologists; BMI: Body Mass Index; CCI: Charlson Comorbidiy Index; HIV: Human Immunodeficiency Virus; N: Number; PA: percutaneous ablation; RLR: robotic liver resection. ^1^ Wilcoxon signed rank test for continuous variables and Fisher’s exact test for categorical variables.

**Table 2:** Liver function and hepatocellular carcinoma (HCC) characteristics (*N* = 60).

Variable	Level	PA (*N* = 36)	RLR (*N* = 24)	*p*-Value ^1^
Ishak fibrosis score, *N* (%)	F0 (none to moderate fibrosis)	14 (39)	6 (25)	0.40
	F1 (severe fibrosis or cirrhosis)	22 (61)	18 (75)	
Portal hypertension, *N* (%)	No	13 (36)	10 (42)	0.79
	Yes	23 (64)	14 (58)	
Esophageal varices, *N* (%)	No	14 (39)	13 (54)	0.30
	Yes	22 (61)	11 (46)	
PLT (migl/mmc), *N* (%)	<100.000	22 (61)	9 (38)	0.11
	≥100.000	14 (39)	15 (63)	
Bilirubin (mg/dl), *N* (%)	≤1	17 (47)	19 (79)	0.017
	>1	19 (53)	5 (21)	
Creatinine (mg/dl), *N* (%)	≤1.4	36 (100)	23 (96)	0.40
	>1.4	0 (0)	1 (4)	
Albumin (g/dl), *N* (%)	<3.5	10 (28)	4 (17)	0.37
	≥3.5	26 (72)	20 (83)	
INR, *N* (%)	≤1.25	19 (53)	18 (75)	0.11
	>1.25	17 (47)	6 (25)	
AFP (ng/mL.), median (min–max)		6.1 (1.5–497.9)	4.3 (1.2–1063.0)	0.34
MELD score, median (min–max)		9 (6–18)	9 (6–17)	0.38
CTP score, *N* (%)	A	28 (78)	19 (79)	1.00
	B	7 (19)	5 (21)	
	C	1 (3)	0 (0)	
ALBI score, *N* (%)	1	9 (25)	15 (63)	0.012
	2	21 (58)	8 (33)	
	3	6 (17)	1 (4)	
Tumor size (mm), median (min-max)		19.5 (12.0–30.0)	20.0 (10.0–30.0)	0.35
Tumor size (mm), *N* (%)	≤20	24 (67)	13 (54)	0.42
	>20	12 (33)	11 (46)	
Subcapsular HCC, *N* (%)	No	20 (56)	6 (25)	0.033
	Yes	16 (44)	18 (75)	
Tumor location, *N* (%)	S1	2 (6)	0 (0)	0.25 ^2^
	S2	1 (3)	3 (13)	
	S3	2 (6)	3 (13)	
	S4	6 (17)	4 (17)	
	S5	7 (19)	3 (13)	
	S6	8 (22)	7 (29)	
	S7	4 (11)	1 (4)	
	S8	6 (17)	3 (13)	
BCLC, *N* (%)	Stage 0	14 (39)	8 (33)	0.79
	Stage A	22 (61)	16 (67)	

AFP: alpha-fetoprotein; ALBI: Albumin-Bilirubin; BCLC: Barcelona Clinic Liver Cancer; HCC: Hepatocellular Carcinoma; INR: International Normalized Ratio; MELD: Model for End-stage Liver Disease; PLT: platelets count. ^1^ Wilcoxon signed rank test for continuous variables and Fisher’s exact test for categorical variables. ^2^ Fisher’s exact test was calculated considering “tumor location” in 3 levels: S2-S3-S4 vs. S5-S6 vs. S1-S7-S8.

**Table 3:** Outcomes (*N* = 60).

Variable	Level	PA (*N* = 36)	RLR (*N* = 24)	*p*-Value ^1^
In-hospital stay (days), median (min–max)		2 (1–6)	4 (1–18)	<0.001
Max bilirubin (mg/dL), median (min–max)		1.45 (0.33–9.88)	1.41 (0.52–9.17)	0.59
Max INR, median (min–max)		1.29 (0.77–1.92)	1.35 (1.07–2.11)	0.12
Max creatinine (mg/dL), median (min–max)		0.86 (0.59–1.29)	0.79 (0.52–2.32)	0.37
Morbidity, *N* (%) ^2 3^	Clavien 0	29 (81)	15 (63)	0.15
	Clavien > 0	7 (19)	9 (38)	
	Clavien 0	29 (81)	15 (63)	0.34
	Clavien I–II	6 (17)	8 (33)	
	Clavien III–IV	1 (3)	1 (4)	
Liver failure, *N* (%)	No	36 (100)	23 (96)	0.40
	Yes	0 (0)	1 (4)	
Readmission within 30 days, *N* (%)	No	34 (94)	23 (96)	1.00
	Yes	2 (6)	1 (4)	

PA: percutaneous ablation and RLR: robotic liver resection. ^1^ Wilcoxon signed rank test for continuous variables and Fisher’s exact test for categorical variables. ^2^ Clavien I and II: 6 in PA (1 patient with gastroparesis, vomit, and naso-gastric tube (NGT) placement; 3 patients with fever; 1 patient with hematoma and fever, and 1 patient with fever and high transaminase levels) and 8 in RLR (3 patients with ascites, 1 patient with pneumothorax, 2 patients with fever, 1 patient with grade B post-hepatectomy liver failure, and 1 patient with pneumonia). Clavien III and IV: 1 in PA (post-procedure subcapsular hematoma and laceration that required arterial embolization and transfusion) and 1 in RLR (abdominal abscess). ^3^ *P*-value of the Fisher’s exact test in Clavien I and II vs. Clavien 0: 0.21. *P*-value of the Fisher’s exact test in Clavien III and IV vs. Clavien 0: 1.00. Note: both patients with Clavien III and IV had an ALBI score = 1. In a logistic model with the event “Clavien > 0” and “Procedure” (PA vs. RLR) and “ALBI score” as covariates, the *p*-value of the procedure was 0.084.

**Table 4:** Outcomes by RLR type of resection (*N* = 60).

Variable	Level	PA (*N* = 36)	Segmentectomy (*N* = 8)	Wedge (*N* = 16)
In-hospital stay (days), median (min–max)		2 (1–6)	4 (1–5)	4 (2–18)
Max bilirubin (mg/dL), median (min–max)		1.45 (0.33–9.88)	1.97 (0.74–4.69)	1.38 (0.52–9.17)
Max INR, median (min–max)		1.29 (0.77–1.92)	1.29 (1.07–1.77)	1.36 (1.14–2.11)
Max creatinine (mg/dL), median (min–max)		0.86 (0.59–1.29)	0.87 (0.59–1.25)	0.76 (0.52–2.32)
Morbidity, *N* (%)	Clavien 0	29 (81)	6 (75)	9 (56)
	Clavien > 0	7 (19)	2 (25)	7 (44)
	Clavien 0	29 (81)	6 (75)	9 (56)
	Clavien I and II	6 (17)	2 (25)	6 (38)
	Clavien III and IV	1 (3)	0 (0)	1 (6)
Liver failure, *N* (%)	No	36 (100)	8 (100)	15 (94)
	Yes	0 (0)	0 (0)	1 (6)
Readmission within 30 days, *N* (%)	No	34 (94)	8 (100)	15 (94)
	Yes	2 (6)	0 (0)	1 (6)

PA: Percutaneous ablation.

## Supplementary Materials

The following are available online at https://www.mdpi.com/2072-6694/12/12/3578/s1, Table S1: Other variables among patients with RLR (*N* = 24), Table S2: Other variables among patients with PA (*N* = 36), Table S3: Overall survival and HCC < 2 cm group, Table S4: Cumulative incidence of recurrence and HCC < 2 cm group, Figure S1: (a) BCLC algorithm and (b) theorical implementation of the BCLC: further validation processes are needed to confirm this proposal, which is currently based only on a best-practice basis.

## References

[1] European Association for the Study of the Liver (2018). Electronic address: Easloffice@easloffice.eu; European Association for the Study of the Liver EASL Clinical Practice Guidelines: Management of hepatocellular carcinoma. J. Hepatol..

[2] Vitali G.C., Laurent A., Terraz S., Majno P., Buchs N.C., Rubbia-Brandt L., Luciani A., Calderaro J., Morel P., Azoulay D. (2016). Minimally invasive surgery versus percutaneous radio frequency ablation for the treatment of single small (≤3 cm) hepatocellular carcinoma: A case-control study. Surg. Endosc..

[3] Groeschl R.T., Gamblin T.C., Turaga K.K. (2013). Ablation for Hepatocellular Carcinoma: Validating the 3-cm Breakpoint. Ann. Surg. Oncol..

[4] Di Sandro S., Benuzzi L., Lauterio A., Botta F., De Carlis R., Najjar M., Centonze L., Danieli M., Pezzoli I., Rampoldi A. (2019). Single Hepatocellular Carcinoma approached by curative-intent treatment: A propensity score analysis comparing radiofrequency ablation and liver resection. Eur. J. Surg. Oncol..

[5] Ciria R., Cherqui D., Geller D.A., Briceno J., Wakabayashi G. (2016). Comparative Short-term Benefits of Laparoscopic Liver Resection: 9000 Cases and Climbing. Ann. Surg..

[6] Rahbari N.N., Garden O.J., Padbury R., Brooke-Smith M., Crawford M., Adam R., Koch M., Makuuchi M., Dematteo R.P., Christophi C. (2011). Posthepatectomy liver failure: A definition and grading by the International Study Group of Liver Surgery (ISGLS). Surgery.

[7] Marrero J.A., Kulik L.M., Sirlin C.B., Zhu A.X., Finn R.S., Abecassis M.M., Roberts L.R., Heimbach J.K. (2018). Diagnosis, Staging, and Management of Hepatocellular Carcinoma: 2018 Practice Guidance by the American Association for the Study of Liver Diseases: Marrero et al. Hepatology.

[8] Karabulut K., Aucejo F., Akyildiz H.Y., Siperstein A., Berber E. (2012). Resection and radiofrequency ablation in the treatment of hepatocellular carcinoma: A single-center experience. Surg. Endosc..

[9] Livraghi T., Meloni F., Stasi M.D., Rolle E., Solbiati L., Tinelli C., Rossi S. (2008). Sustained complete response and complications rates after radiofrequency ablation of very early hepatocellular carcinoma in cirrhosis: Is resection still the treatment of choice?. Hepatology.

[10] Liu P.H., Hsu C.Y., Hsia C.Y., Lee Y.H., Huang Y.H., Chiou Y.Y., Lin H.C., Huo T.I. (2016). Surgical Resection Versus Radiofrequency Ablation for Single Hepatocellular Carcinoma ≤ 2 cm in a Propensity Score Model. Ann. Surg..

[11] Sposito C., Battiston C., Facciorusso A., Mazzola M., Muscarà C., Scotti M., Romito R., Mariani L., Mazzaferro V. (2016). Propensity score analysis of outcomes following laparoscopic or open liver resection for hepatocellular carcinoma. Br. J. Surg..

[12] Cucchetti A., Mazzaferro V., Pinna A.D., Sposito C., Golfieri R., Serra C., Spreafico C., Piscaglia F., Cappelli A., Bongini M. (2017). Average treatment effect of hepatic resection versus locoregional therapies for hepatocellular carcinoma. Br. J. Surg..

[13] Di Sandro S., Bagnardi V., Najjar M., Buscemi V., Lauterio A., De Carlis R., Danieli M., Pinotti E., Benuzzi L., De Carlis L. (2018). Minor laparoscopic liver resection for Hepatocellular Carcinoma is safer than minor open resection, especially for less compensated cirrhotic patients: Propensity score analysis. Surg. Oncol..

[14] Lim C., Osseis M., Lahat E., Doussot A., Sotirov D., Hemery F., Lantéri-Minet M., Feray C., Salloum C., Azoulay D. (2019). Safety of laparoscopic hepatectomy in patients with hepatocellular carcinoma and portal hypertension: Interim analysis of an open prospective study. Surg. Endosc..

[15] Di Benedetto F., Petrowsky H., Magistri P., Halazun K.J. (2020). Robotic liver resection: Hurdles and beyond. Int. J. Surg..

[16] Ban D., Tanabe M., Ito H., Otsuka Y., Nitta H., Abe Y., Hasegawa Y., Katagiri T., Takagi C., Itano O. (2014). A novel difficulty scoring system for laparoscopic liver resection. J. Hepatobiliary Pancreat. Sci..

[17] Di Benedetto F., Tarantino G., Magistri P. (2019). Chasing the Right Path: Tips, Tricks and Challenges of Robotic Approach to Posterior Segments. Hepatobiliary Surg. Nutr..

[18] Magistri P., Guerrini G.P., Ballarin R., Assirati G., Tarantino G., Di Benedetto F. (2019). Improving Outcomes Defending Patient Safety: The Learning Journey in Robotic Liver Resections. Biomed. Res. Int..

[19] Chen P.D., Wu C.Y., Hu R.H., Chen C.N., Yuan R.H., Liang J.T., Lai H.S., Wu Y.M. (2017). Robotic major hepatectomy: Is there a learning curve?. Surgery.

[20] Magistri P., Pecchi A., Franceschini E., Pesi B., Guadagni S., Catellani B., Assirati G., Guidetti C., Guerrini G.P., Tarantino G. (2019). Not just minor resections: Robotic approach for cystic echinococcosis of the liver. Infection.

[21] Von Elm E., Altman D.G., Egger M., Pocock S.J., Gøtzsche P.C., Vandenbroucke J.P. (2007). STROBE Initiative The Strengthening the Reporting of Observational Studies in Epidemiology (STROBE) statement: Guidelines for reporting observational studies. Lancet.

[22] Dindo D., Demartines N., Clavien P.A. (2004). Classification of surgical complications: A new proposal with evaluation in a cohort of 6336 patients and results of a survey. Ann. Surg..

[23] Magistri P., Tarantino G., Guidetti C., Assirati G., Olivieri T., Ballarin R., Coratti A., Di Benedetto F. (2017). Laparoscopic versus robotic surgery for hepatocellular carcinoma: The first 46 consecutive cases. J. Surg. Res..

