# Supplementary material for: Robotic Liver Resection Versus Percutaneous Ablation for Early HCC: Short- and Long-Term Results
Source: Cancers (Basel). 2020 Nov 30;12(12):3578. doi: 10.3390/cancers12123578 (PMC7761404; doi:10.3390/cancers12123578)
Supplement: Supplementary file 1 [file cancers-12-03578-s001.pdf]

Supplementary Materials: Robotic Liver Resection Versus Percutaneous Ablation for Early HCC: Short- and Long-Term Results

Paolo Magistri, Barbara Catellani, Samuele Frassoni, Cristiano Guidetti, Tiziana Olivieri, Giacomo Assirati, Cristian Caporali, Annarita Pecchi, Valentina Serra, Roberto Ballarin, Gian Piero Guerrini, Vincenzo Bagnardi, Stefano Di Sandro and Fabrizio Di Benedetto

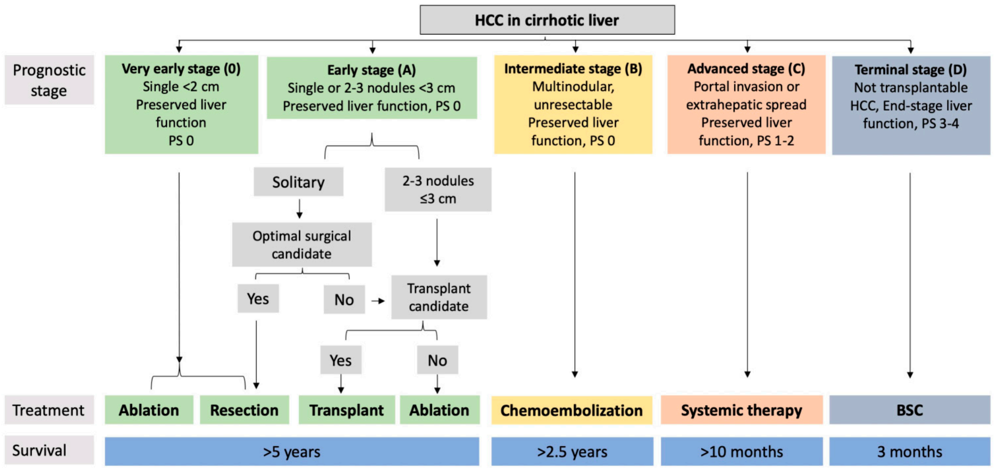

(a)

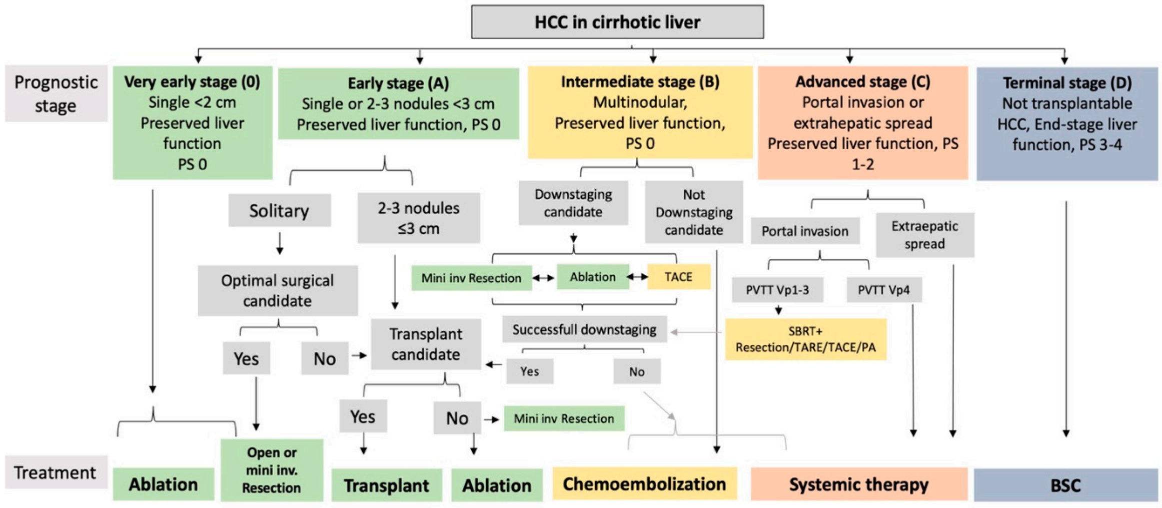

(b)

Figure S1. (**a**) BCLC algorithm; (**b**) theorical implementation of the BCLC: further validation processes are needed to confirm this proposal that is currently based only on a best-practice basis.

Publisher's Note: MDPI stays neutral with regard to jurisdictional claims in published maps and institutional affiliations.

© 2020 by the authors. Licensee MDPI, Basel, Switzerland. This article is an open access article distributed under the terms and conditions of the Creative Commons Attribution (CC BY) license (http://creativecommons.org/licenses/by/4.0/).
